# Supplementary material for: Ceratocarpus arenarius: Botanical Characteristics, Proximate, Mineral Composition, and Cytotoxic Activity
Source: Molecules. 2024 Jan 12;29(2):384. doi: 10.3390/molecules29020384 (PMC10819601; doi:10.3390/molecules29020384)
Supplement: Supplementary file 1 [file molecules-29-00384-s001.zip › molecules-2727203-supplementary.pdf]

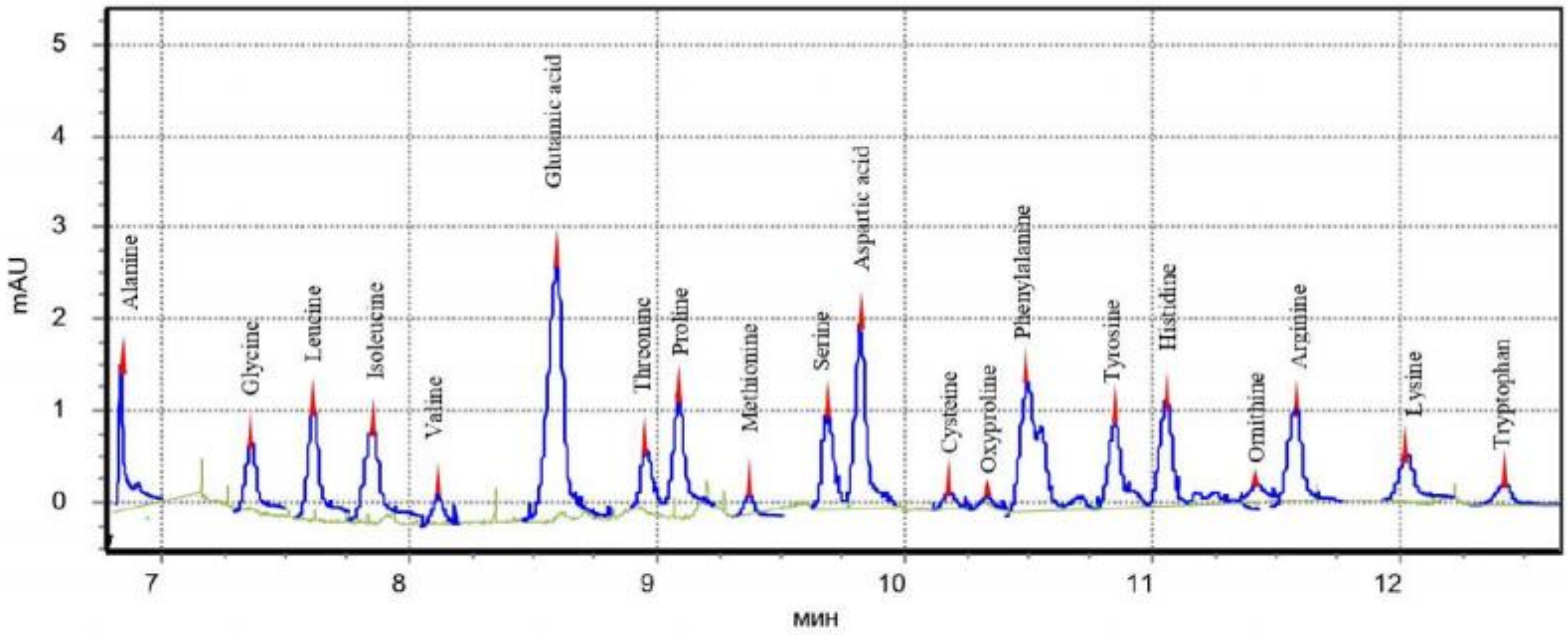

Figure S2. GC/MS Chromatogram of amino acid Ceratocarpus arenarius L.

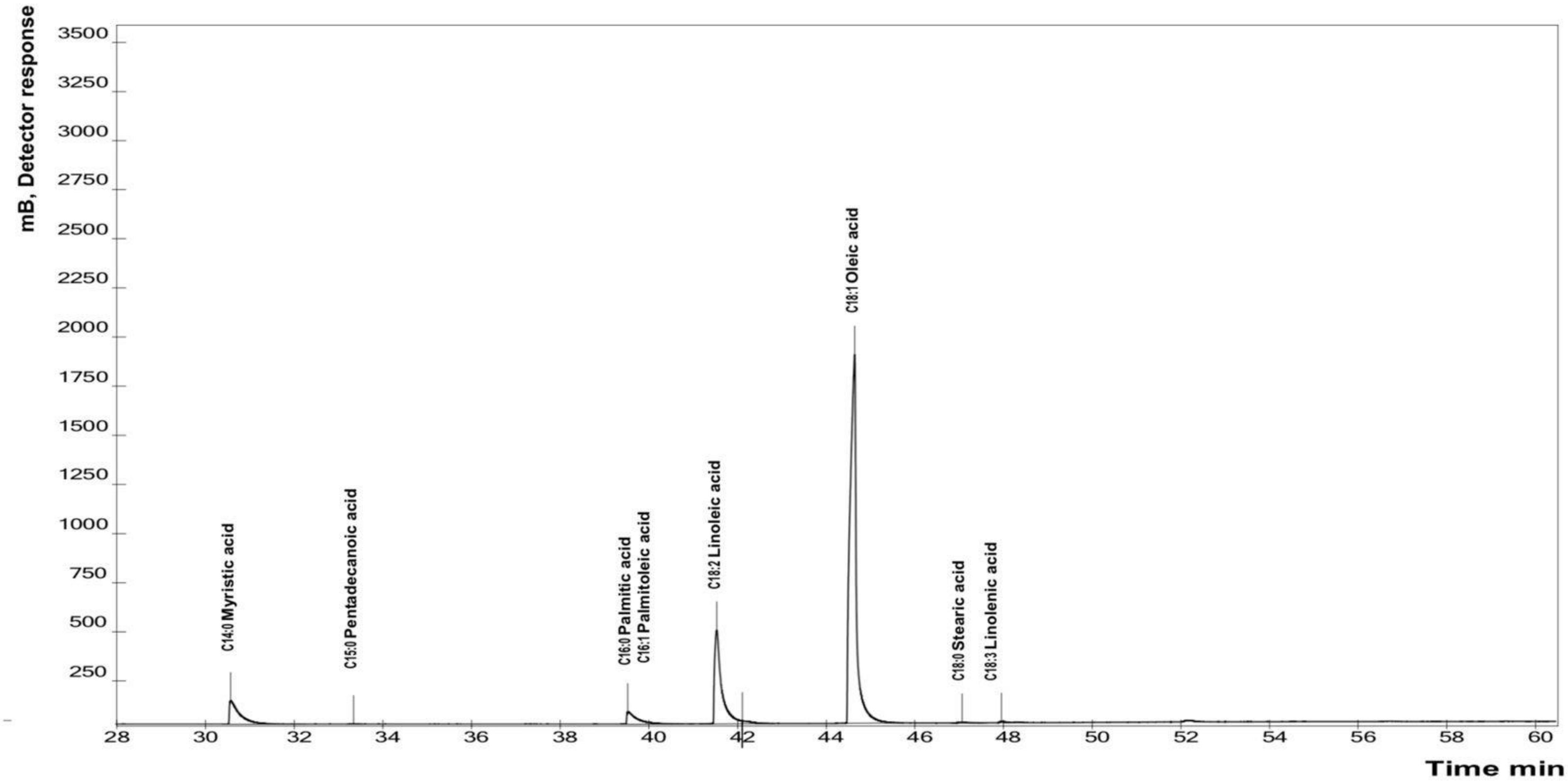

Figure S1. GC/MS Chromatogram of fatty acid *Ceratocarpus arenarius L.*
